# Self-Determination in Global Health Practices – Voices from the Global South

**DOI:** 10.5334/aogh.4162

**Published:** 2024-02-28

**Authors:** Maureen Kesande, Jane Jere, Sandra I. McCoy, Abel Wilson Walekhwa, Bongekile Esther Nkosi-Mjadu, Eunice Ndzerem-Shang

**Affiliations:** 1 University of California, Berkeley, School of Public Health, 2121 Berkeley Way, Room 2220 Berkeley, CA 94720-7360, US; 2 Diseases Dynamics Unit, Department of Veterinary Medicine, University of Cambridge, Madingley Road, Cambridge CB3 0ES, UK

**Keywords:** Decolonization, Global Health, Education, Research, Collaboration, Equity

## Abstract

Despite the commendable progress made in addressing global health challenges and threats such as child mortality, HIV/AIDS, and Tuberculosis, many global health organizations still exhibit a Global North supremacy attitude, evidenced by their choice of leaders and executors of global health initiatives in low- and middle-income countries (LMICs). While efforts by the Global North to support global health practice in LMICs have led to economic development and advancement in locally led research, current global health practices tend to focus solely on intervention outcomes, often neglecting important systemic factors such as intellectual property ownership, sustainability, diversification of leadership roles, and national capacity development. This has resulted in the implementation of practices and systems informed by high-income countries (HICs) to the detriment of knowledge systems in LMICs, as they are deprived of the opportunity to generate local solutions for local problems.

From their unique position as international global health fellows located in different African countries and receiving graduate education from a HIC institution, the authors of this viewpoint article assess how HIC institutions can better support LMICs. The authors propose several strategies for achieving equitable global health practices; 1) allocating funding to improve academic and research infrastructures in LMICs; 2) encouraging effective partnerships and collaborations with Global South scientists who have lived experiences in LMICs; 3) reviewing the trade-related aspects of intellectual property Rights (TRIPS) agreement; and 4) achieving equity in global health funding and education resources.

## Background

Global health is the area of study, research, and practice that prioritizes improving health and achieving equity in health for all people worldwide [1]. Chen, Li et al. argue that global health encompasses multi-country and global projects or studies conducted in a local area that is either framed with a global perspective, intended to address an issue with global impact, and that seek global solutions to issues through frameworks, strategies, policies, laws, or regulations [1].

Global health issues are too vast for any single country or continent to tackle. Recent outbreaks of infectious diseases such as Ebola virus, Zika virus, Rift Valley fever, Middle East respiratory syndrome (MERS), COVID-19, and others demonstrate the continuing impact of the transborder spread of such diseases. This has led to an increase in opportunities to study and practice globally, piquing researchers’ interests and leading to the rapid expansion of global health research and education.

According to the decolonizing global health toolkit, the primary aims of decolonizing global health practices are: to achieve equitable collaborations, center projects around local priorities, and promote respectful, collaborative interactions and language in all communications [2]. However, for reasons such as fear of retaliation or feelings of powerlessness, the voices of global health institutions and practitioners based in the Global South have largely been absent amidst the calls for decolonization [3], especially during the COVID-19 global pandemic, at a time when the world most needed to hear from them. This has led some students and researchers, including those of African origin working in Global North institutions, to question if decolonizing global health is possible, or if it requires the steering of institutions based in the Global South with headquarters in the Global North to be put in the hands of leaders from the Global South [4,5].

The authors of this viewpoint article share their thoughts on how the support from HICs has contributed to improving public health practices in LMICs while simultaneously creating disparities in leadership, intellectual property (IP) rights ownership, authorship recognition, and graduate exchange programs that require urgent attention in a bid to decolonize global health. They suggest actionable steps to bridge these gaps.

## Discussion for the attainment of decolonized global health education and research – Fellows perspective

### (i) Effective global health support in LMICs

Over the years, strategic North-South partnerships have been established to meet global health policies such as the Millennium and Sustainable Development Goals. Many institutions have provided technical assistance to LMICs through fellowships that support postdoctoral fellows and graduate students in long-term, hands-on mentored research [6]. However, the value added to the development and prosperity of the Global South countries by these contributions remains uncertain. What are the economic benefits and outcomes of these investments to the beneficiaries’ countries of origin? Despite the efforts to invest in capacity building, less attention has been paid to upgrading pharmaceutical manufacturing, Good Manufacturing Practice (GMP) facilities, pilot production structures, and systems in LMICs to effectively utilize the newly acquired skills. For instance, numerous Global South graduate students undergo training in the development of vaccines, diagnostics, and surveillance tools development in highly specialized laboratories situated in the Global North institutions. However, upon their return, the absence of adequate infrastructure hinders their ability to apply the acquired skills, leaving them with theoretical knowledge, and their skills diminish over time. Effective support for world-class infrastructure and training is necessary to address this gap, particularly for younger researchers. A strategic direction in targeted global health efforts is to invest in rebuilding foundational infrastructure systems in LMICs, in addition to supporting individual careers of global health researchers in LMICs. However, HICs may face significant challenges and be reluctant to invest in such infrastructure in LMICs. The reluctance of foreign investors to invest in Africa stems from the relatively high level of uncertainty which manifests as political instability, macroeconomic instability, and lack of transparency in policies [7]. In the face of these challenges, policymakers in LMICs must foster an environment that is favorable for long-term investments in infrastructure by establishing robust institutions that restrict unilateral policy changes by governments, mitigating political risks. High-income countries and private entities that are engaged in infrastructure investment should prioritize managing risks in African projects instead of completely avoiding them [8]. Collaborative efforts are required at national, regional, and international levels to attract investment inflows into Africa [7]. This approach will foster sustainable development and long-term prosperity for the beneficiaries’ country of origin.

### (ii) Global partnerships and their impact

Partnerships between the Global South and North should yield tangible gains in addressing global health challenges, such as HIV/AIDS, tuberculosis, and tropical diseases, with intellectual contributions recognized and treated equally [9]. However, in reality, the Global North manages and controls production lines, industries, and intellectual property, forcing Global South collaborators to import the final products that they contributed to through research and clinical trials. Such partnerships result in imbalances in authorship and benefit the better-resourced partner [10,11]. A lived example is the practice between Uganda’s oldest research institutions – Uganda Virus Research Institute (UVRI) and Makerere University, Uganda Cancer Institute, and their Global North partners. Although specimens such as blood and body tissues are obtained during epidemic responses there, Uganda seldom benefits from intellectual property contributions partly due to the challenges faced by many of its institutions in effectively using intellectual property policies and systems as evidenced by low levels of intellectual property awareness, registration, or enforcement [12]. The Industrial Property Act of 2014, as stated in Part III, Section 8(3) of the Act, excludes discoveries, scientific theories, diagnostic, and pharmaceutical products from patent protection, which can directly hinder scientific research in the least developed countries [13,14]. According to Uganda’s May 2019 Ministry of Justice and Constitutional Affairs’ National Intellectual Property Policy, the level of registration of intellectual property rights in Uganda remains low, compared to other countries; for example, only eight patents were granted in 2015, compared to 207 in Kenya; 7,552 in South Africa; 16,135 in Mexico; 25,956 in Australia; 65,965 in Germany; and 578,802 in the USA the same year [12].

Research grants channeled through institutions in HICs to scientists and researchers in LMICs often fail to strengthen project coordination capacity and expertise in LMIC institutions because experts from the funding organization coordinate the projects. Although the establishment of the first COVID-19 mRNA vaccine technology transfer hub in South Africa, with the assistance of the World Health Organization (WHO), brought hope for growth in research and development in the Global South, challenges remained in scaling up efforts due to intellectual property barriers that prevent full operationalization of the messenger Ribonucleic Acid (mRNA) technology [15]. Nonetheless, the project aims to advance global efforts to build vaccine development and manufacturing capacity in Africa, which would promote self-determination and contribute to decolonizing global health [16].

The Trade-Related Aspects of Intellectual Property Rights (TRIPS) Agreement currently restrains the patenting of newly discovered biological research products from many Global South countries, primarily in Africa [17]. In June 2022, the World Trade Organization’s (WTO) 12th Ministerial Conference agreed on measures to diversify global production of COVID-19 vaccines and on ways to build resilience to future pandemics; members will have greater scope over the next five years to override the exclusive effect of patents through a targeted waiver that addresses specific problems identified during the pandemic, especially facilitating and streamlining COVID-19 vaccine exports [18]. In May 2024, the World Health Assembly is expected to consider a draft of the agreement that has been referred to as the pandemic treaty in which countries have to agree on how to better respond to future pandemics [19]. Such continued revisions of the TRIPS agreement and dialogue among the WHO member nations are needed, with attention to the interests of Global South countries, thereby motivating researchers, and advancing technology transfer, ultimately contributing to a sustainable world.

The COVID-19 vaccine procurement demonstrated that countries with the capacity to manufacture or procure vaccines prioritize their citizens’ and countries’ interests over global health interests, leaving incapacitated Global South countries in limbo [17]. Fair partnerships are critical for the Global South to make headway in addressing global health challenges, as shown by the case study of the COVID-19 vaccine, which explores some of the factors explaining why Africa finds itself at the back of the queue for access to life-saving therapeutics [20].

### (iii) Funding, funder perspective, and requirements

Global Health research funding agencies in HICs tend to favor researchers with previous experience in grant management, which is often limited to those trained and practicing in HICs [21]. This leads to biased allocation of funds, as researchers in low-resourced institutes in LMICs often lack the necessary training and time to write grant applications [21]. The rejection of grant proposals without feedback or with limited feedback further hampers research capacity building and contributes to brain drain in LMICs [21]. To address this, academic institutions should encourage researchers from the Global South to support their home countries through mentorship programs, affordable consultancy services, grant writing, and partner extension to foster technology and knowledge transfer. Funding agencies could enforce this by dedicating a certain percentage of research funding to local leadership.

Sustainable funding bodies could also be created within LMICs. For example, the African Population and Health Research Center (APHRC) is a progressive African-led non-profit global research center that strategically partners and builds relationships with key decision-making bodies at the national, regional, and global levels. It encompasses engagements with different entities to ensure contextual, relevant, and localized knowledge as a driver of change, by nurturing African research leadership.

To ensure equity and strong representation in global health, research investments from HICs must contribute to the improvement of academic infrastructures in LMICs [22]. Redistributing funding away from HICs is also proposed as a means to address the imbalance of power in global health organizations, as evidenced by the lack of representation from developing countries in institutions like the International Monetary Fund (IMF) and the World Bank [23]. Such imbalances lead to the recruitment of LMIC talent by HICs, resulting in weakened health systems and a loss of opportunities to generate local solutions to local problems [23].

Establishing think tanks, conferences, and research groups in Global South countries can promote knowledge exchange, dissemination of Global South data, and shared learning among scientists in the Global South, contributing to sustainable development and decolonizing global health. The Data Science for Health Discovery and Innovation in Africa (DS-I Africa) Initiative funded by NIH serves as a notable example. It aims to contribute to the development of expertise among African scientists and create a pan-continental network with broad academic, public, private, and industry partnerships to provide local solutions to countries’ most immediate public health problems [24]. The authors encourage the provision of more opportunities for funding for global health education and research in LMICs, led by Indigenous experts in various fields, without requiring researchers from HICs to lead and take ultimate ownership of the activities.

### (iv) Equity in global health education and resources

To involve national academic leaders and institutions in collaborative education design, the authors propose adopting the Virtual Roundtable for Collaborative Education Design (ViRCoED) model developed and piloted by Sbaiti et al [25]. Their approach recognizes the importance of including stakeholders with lived experiences in curriculum development to ensure relevance to the context being studied. By involving external collaborators with such experiences, deeper engagement with global health issues and a more representative body of knowledge can be achieved.

Global health, at its core, aims to achieve health equity for all people [26]. Given this principle, it is necessary to confront the power imbalances and historical legacy of colonialism that continue to shape global health partnerships and educational programs between HICs and LMICs. To achieve this, students must have a strong foundation in understanding health equity and practical strategies for achieving it [27].

Many institutions have developed structured curricula for global health exchange programs, but these exchanges must be more reciprocal and equitable between countries, communities, and organizations. Currently, more HIC students participate in educational and experience-sharing opportunities in LMICs than vice versa, highlighting the inequitable funding structures within institutions [11].

Mbaye et al. [28] and Boum et al. [29] argue that research partnerships with LMICs tend to favor HIC counterparts. Funders often define research priorities and determine where and how findings are published, limiting local impact and utilization. Eichbaum et al. [11] suggest addressing this issue by recruiting more faculty from the Global South in Global North institutions, investing in academic infrastructures in LMICs, and involving LMIC national academic leaders and institutions in global health education. Since recruitment from the South to the North would contribute to brain drain, the authors suggest HIC academic institutions have partnerships with LMIC institutions to create endowed faculty positions with joint appointments. Abimbola and Pai [10] recommend democratizing knowledge platforms, making high-impact Western journals accessible in the Global South, and valuing academic institutions in the Global South as highly as those in the Global North, with a focus on serving disadvantaged populations in both settings. They also call for mutual learning, not dependence, making global health degrees accessible to those who need them the most, and prioritizing teaching conducted by those on the front lines. Binagwaho et al. [30] propose investing in capacity strengthening for existing global health institutions in LMICs to challenge the notion that knowledge only flows from HICs to LMICs, which reinforces a narrative of white supremacy.

## Conclusions

Despite the many progressive efforts and recommendations by experts to “decolonize global health and global health education,” there are undoubtedly still many barriers to achieving this. These include overemphasizing research results and ignoring issues like intellectual property ownership, research investment and sustainability, development of national research agendas and capacity, and the diversification of leadership roles in partnerships between the Global North and South.

Attempts to decolonize global health must focus on eliminating the root cause, which is the white supremacy mindset. Only then would effective global health support in LMICs be realized. That would include support for world-class infrastructure and training of younger researchers. Adoption of the ViRCoED model, which will see the inclusion of stakeholders with lived experiences in curriculum development to ensure relevance to the context being studied. Confrontation of power imbalances, and recruitment of more faculty from the Global South in Global North institutions. Effective partnerships that promote self-determination through the strengthening of project coordination capacity and provision of expertise to LMICs. Urgent review of the TRIPS agreement on intellectual property protection, which would advance technology transfer, as many Global South countries will freely patent newly discovered biological research products, increasing sustainability.

A truly collaborative and equitable global health education will be achieved if academic, research, and training institutions with an underrepresentation of LMIC faculty, staff, and students set targets and take deliberate steps to diversify their approach. This will ensure there is more equitable representation in publications and leadership roles, a major step in the pursuit of meaningful partnerships. Funding agencies should be diverse and include LMIC representatives. They should encourage researchers from LMICs to support their home countries through mentorship programs, affordable consultancy services, grant writing, and partner extension to foster technology and knowledge transfer. Bias in research funding and publications should be eliminated, making global health programs accessible to scholars from LMICs to increase the number of trained professionals, strengthening their education and research capacity.

In addition to the recommendations provided, the authors envision a global ecosystem where they are players through continental and international partnerships; as well as philanthropy to fund innovation where donors fund incremental changes in LMICs’ research.

## Data Accessibility Statement

The raw data that we used to compile this manuscript is available on request. Please contact the corresponding author.
